# Prevalence of healthy lifestyles against cancer in Spanish women

**DOI:** 10.1038/s41598-019-47180-x

**Published:** 2019-07-23

**Authors:** María José Toribio, Virginia Lope, Adela Castelló, Dolores Salas, Carmen Vidal, Nieves Ascunce, Carmen Santamariña, Pilar Moreo, Carmen Pedraz-Pingarrón, Carmen Sánchez-Contador, Nuria Aragonés, Beatriz Pérez-Gómez, Marina Pollán

**Affiliations:** 1 Servicio de Medicina Preventiva y Gestión de Calidad, Hospital General Universitario Gregorio Marañón, Madrid, Spain; 2 Cancer and Environmental Epidemiology Unit, National Center for Epidemiology, Carlos III Institute of Health, Madrid, Spain; 3 Consortium for Biomedical Research in Epidemiology & Public Health, CIBERESP, Madrid, Spain; 4 General Directorate Public Health, and FISABIO, Valencia, Spain; 5 Cancer Prevention and Control Unit, Catalan Institute of Oncology (ICO), Barcelona, Spain; 6 Navarra Breast cancer Screening Program, Public Health Institute, Pamplona, Spain; 7 Galicia Breast Cancer Screening Program, Regional Authority of Health, Galicia Regional Government, Corunna, Spain; 8 Aragon Breast Cancer Screening Program, Health Service of Aragon, Zaragoza, Spain; 9 Castile-Leon Breast Cancer Screening Program, General Directorate Public Health SACYL, Burgos, Spain; 10 Balearic Islands Breast Cancer Screening Program, Health Promotion for Women and Childhood, General Directorate Public Health and Participation, Regional Authority of Health and Consumer Affairs, Balearic Islands, Palma, Spain; 11 Epidemiology Section, Public Health Division, Department of Health of Madrid, Madrid, Spain

**Keywords:** Risk factors, Cancer prevention, Cancer epidemiology

## Abstract

Modifying behavior towards healthier lifestyles could prevent a significant number of malignant tumors. We evaluated the prevalence of healthy habits against cancer in Spanish women free of this disease, taking as a reference the recommendations for cancer prevention included in the European Code Against Cancer (ECAC), and we explored the characteristics associated with it. Our population comprised 3,584 women recruited in a population-based cross-sectional study carried out in 7 breast cancer screening programs. Information was directly surveyed and used to calculate a score based on ECAC recommendations referred to bodyweight, physical activity, diet, breastfeeding, tobacco, alcohol and hormone replacement therapy use. The degree of adherence was estimated with a score that evaluated null (0 points), partial (0.5 points) and full adherence (1 point) of each specific recommendation. Associations were explored using binary and ordinal logistic regression models. The median score was 5.7 out of 9 points. Recommendations with lower adherence were those related to intake of red/processed meat and foods high in salt (23% of total adherence), physical activity (24%) and body weight (29%), and recommendations with greater adherence where those related to hormone replacement therapy use (91%), vegetable intake (84%), alcohol (83%) and tobacco (61%). Overall adherence was better among older women, parous women, and in those living in rural areas, and worse among women with higher caloric intake. These recommendations should be evaluated periodically. Screening programs can be an appropriate place to disseminate this information.

## Introduction

Cancer is the second leading cause of death globally. It was responsible for 9.6 million deaths in 2018 accounting for one of every 6 deaths^1^. In Spain it was the second cause of mortality in women, accounting for 20% in 2015^2^. In comparison with other European countries, the incidence of cancer in Spanish women (227.1 cases per 100,000) is below the European mean (253.3 per 100,000). However, it is the second country with the lowest mortality due to this cause, with 65.9 deaths per 100,000 in 2018^3^. Despite the stabilization of breast cancer incidence, there has been an increase in colorectal and tobacco-related cancers, and geographic differences in incidence rates have been described^4^. Malignant tumors also imply a substantial economic burden across European Union^5^. It has been estimated that the health-care cost of cancer in Spain was 4114 million euros in 2009, equivalent to €102 per citizen. It accounted for 4% of total Spanish health-care expenditure, and the 0.86% of gross domestic product. Breast cancer had the greatest overall economic burden^5^.

Cancer is a complex illness resulting from the interaction between genes and environmental factors such as tobacco^6–8^, obesity/overweight^9–12^, unhealthy diets^10,12,13^, physical inactivity^10,12,14,15^, alcohol intake^8,10,12,16^, certain environmental and occupational exposures^6,17,18^, ionizing radiation^19,20^, several infectious agents^21^, and hormone therapy^22,23^. A significant number of cases could be prevented avoiding or reducing exposure to these risk factors^24^. Several studies have analyzed adherence to cancer prevention guidelines and their relationship to this disease^25–27^, but have not analyzed the sociodemographic and lifestyle factors influencing such adherence^28^. In Spain, although trends for many risk factors -such as alcohol consumption or healthier diets- have improved in recent years, the prevalence of tobacco consumption among men remains high; there has been an upward trend in smoking among women aged 45–64 years, and physical inactivity has conditioned an increase in obesity figures in the general population^29^.

To emphasize the importance of cancer prevention, the International Agency for Research on Cancer (IARC) published in 2014 the new edition of the European Code Against Cancer (ECAC), with 12 recommendations regarding primary and secondary prevention: (1) Do not smoke; (2) Support smoke-free policies; (3) Be a healthy body weight; (4) Be physically active; (5) Have a healthy diet (three recommendations are included here: 5.1 eat plenty of whole grains, pulses, vegetables and fruits; 5.2 limit high-calorie foods and avoid sugary drinks; 5.3 avoid processed meat and limit red meat and foods high in salt); (6) Limit your alcohol intake; (7) Avoid too much sun; (8) Protect yourself against cancer-causing substances in your workplace; (9) Take action to reduce high radon levels; (10) Breastfeed your baby and limit use of hormone replacement therapy (HRT); (11) Ensure your children take part in vaccination programmes for hepatitis B (newborns) or human papillomavirus (girls); (12) Take part in cancer screening programmes for bowel cancer (men and women), breast and cervical cancer (women)^30,31^.

Our objective is to evaluate the prevalence of healthy habits against cancer in women who attend breast cancer screening programs in Spain, taking as a reference of good practices the recommendations of ECAC related to smoking, obesity, diet, alcohol, breastfeeding and HRT use. We also try to explore what sociodemographic and lifestyle factors are associated. This information can be useful to define priorities and public health actions with the purpose of reducing the burden of cancer in the population and the inequalities between different Spanish geographical areas.

## Materials and Methods

### Study population

DDM-Spain study (Determinants of Mammographic Density in Spain) is a cross-sectional multicenter study that recruited, between October 2007 and July 2008, 3,584 healthy women, aged 45 to 68 years, from 7 specific screening centers within the Spanish breast cancer screening programme network. In Spain, all women aged 50 to 69 years, regardless of their nationality or legal status, are screened under these government-sponsored population-based programs every 2 years (in some regions women aged 45 to 49 are also included). Attendance rates range from 62.9% to 88.0% of its target population, depending on the region. Seven screening centers located in Aragon, Balearic Islands, Castile-Leon, Catalonia, Galicia, Navarre, and Valencian Region participated in DDM-Spain. The recruiting centers contacted all screening attendants by telephone and invited them to participate in the study. Those who accepted were interviewed at their screening center the day scheduled for their mammogram, shortly after the first telephone contact. As a measure to minimize erroneous information, women were re-contacted by phone to corroborate inconsistent or unclear data after the interview. Furthermore, a subsample of 150 women were re-interviewed to test the consistency of the information reported, particularly regarding their diet, given the difficulty inherent to food frequency questionnaires, with very good agreement between the first and second questionnaires^32^. The overall participation rate was 74.5% (64.7%–84.0%).

The study was approved by the Research Ethics Committee of the Carlos III Institute of Health, and all participants signed an informed consent form. All research was conducted in accordance with relevant guidelines and regulations. Further information about the DDM-Spain study can be consulted elsewhere^33–35^.

### Study variables

Structured face-to-face questionnaires were administered by trained interviewers to collect data about women’s sociodemographic, reproductive, and lifestyle habits. The survey included a 117-item validated semi quantitative food frequency questionnaire that collected the eating habits of the previous 12 months^36,37^. In addition, an anthropometric examination of all participants was conducted following standardized procedures, and using the same scale, stadiometer and measuring tape models in all study centers.

We elaborated an index based on the following 9 ECAC recommendations (including diet sub-recommendations) for primary prevention of cancer: (1) Do not smoke; (2) Be a healthy body weight; (3) Be physically active; (4) Eat plenty of whole grains, pulses, vegetables and fruits; (5) Limit high-calorie foods and avoid sugary drinks; (6) Avoid processed meat, limit red meat and foods high in salt; (7) Limit your alcohol intake; (8) Breastfeed your baby; and (9) Limit HRT use. We could not include other ECAC recommendations due to lack of information. A maximum score of 1 was assigned when the recommendation was fully met, an intermediate value of 0.5 when the recommendation was not far from being met and 0 points otherwise (see Supplementary Table S1). Given their heterogeneity, specific recommendations related to diet were evaluated separately, using the cut-off points proposed by a previous study^25^.

The final score was obtained as the sum of the individual scores. Therefore, the ECAC score ranged from 0 (none of these recommendations was met) to 9 (all of them were fully met). Taking into account the distribution of the score, three categories of adherence were considered: low adherence (<5), moderate adherence (5–<6.5) and high adherence (≥6.5). These cut-off points were determined as tertiles of each individual score.

The following characteristics were evaluated as possible determinants of adherence to the ECAC recommendations: age, menopausal status (defined as absence of menstruation in the last 12 months), geographic area, self-reported socioeconomic level at the time of the interview and at birth, educational level, ethnicity, civil status, town-size of residence, occupational status, frequency of eating out, having to take care children and/or elderly people at home, family history of cancer, and reproductive factors (number of miscarriages/abortions and number of deliveries).

### Statistical analyses

Descriptive analyses of women’s characteristics were performed using means and standard deviations for continuous variables and absolute figures and percentages for categorical variables. Significant differences were tested using Pearson chi-square for categorical variables and Student’s t-test for continuous variables.

The association between the three categories of adherence, as a measure of overall compliance to ECAC recommendations, and sociodemographic and lifestyle characteristics, was evaluated using ordinal logistic regression models. First of all, a minimum adjustment model was used, for each factor including only age and place (location of the screening center) as possible confounders. Then, a final multivariate model was fitted considering all those factors associated with the score in the previous model (p < 0, 10) (namely, age, screening center, educational level, size of residence town, eating out, civil status, and number of deliveries).

The effect of these factors on the adherence to individual ECAC recommendations was also evaluated using multivariate logistic regression models. A separate model was fitted for each specific recommendation, considering the same determinants included in the multivariate model mentioned before. In the case of the geographical location of the screening center, the average value of all of them was used as a reference. These models were also adjusted for the overall adherence to the other recommendations, which were obtained by adding up the scores of all individual recommendations, except the one under study.

Analyses were performed using STATA/MP 12.0 software.

## Results

**Figure 1:**
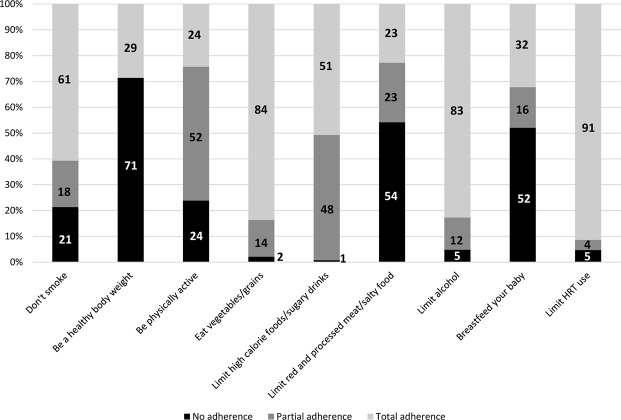
Degree of adherence to European Code Against Cancer recommendations.

Of the 3584 women recruited, we analyzed those from which we obtained complete data for the variables age and location of the screening center (3550). The distribution of sociodemographic and life-style characteristics of these women is shown in Table 1. Mean age was 56 years. Most women were postmenopausal (77%), of medium socioeconomic status (71%), of low social status at birth (58%), with secondary education (56%), Caucasian (99%), and married (80%). Most of them lived in small cities (75%) and were active workers or had worked in the past (86%). Only 8% of women lived with people who needed care at home and 48% reported eating out at least 2 times/month (on average, these women ate out between 5 and 6 times per month). Only 9% of them were nulliparous, 28% had more than two children and 21% reported one or more miscarriages/abortions. Regarding family history of cancer, a substantial proportion reported having a first or second degree relative with a diagnosis of cancer at any location (46%). The overall score ranked between 1 and 9 with a mean of 5.71. 33% of the women showed low adherence to the ECAC recommendations. This percentage was 50% for moderate adherence and 17% for high adherence. On average, women with higher adherence were older, while low adherence was more prevalent among those women with higher educational level, single, nulliparous, those who lived in more populated towns, those gainfully employed, and in those who reported frequently eating out and consumed more calories (Table 1). In relation to individual recommendations, adherence was very high regarding the limitation of HRT use (91%), the frequent intake of grains, pulses, vegetables and fruits (84%) and low alcohol consumption (83%), and the adherence was lower for the low consumption of processed meat, red meat and foods high in salt (23% of total adherence) and for the recommendation of physical activity (24%) (Fig. 1).

**Table 1:** Characteristics of women participating in the DDM-Spain study*.

Variables	Total	Low adherence^a^	Moderate adherence^a^	High adherence^a^	p^b^
		(<5)	(5–<6.5)	(≥6.5)	
Age, Mean (SD)	56.2 (5.5)	55.2 (5.4)	56.6 (5.5)	56.9 (5.3)	<0.001
Menopausal status, N (%)					
Postmenopausal	2738 (77.1)	879 (74.1)	1381 (78.5)	478 (79.3)	0.01
Pre/perimenopausal	812 (22.9)	308 (25.9)	379 (21.5)	125 (20.7)	
Social status, N (%)					
Low	850 (24.1)	265 (22.4)	441 (25.2)	144 (23.9)	0.46
Medium	2509 (71.0)	860 (72.6)	1218 (69.7)	431 (71.6)	
High	176 (5.0)	60 (5.0)	89 (5.1)	27 (4.5)	
Social status at birth, N (%)					
Low	2018 (57.5)	679 (57.7)	998 (57.5)	341 (57.3)	0.76
Medium	1344 (38.3)	441 (37.5)	672 (38.7)	231 (38.8)	
High	146 (4.2)	56 (4.8)	67 (3.8)	23 (3.9)	
Educational level, N (%)					
Primary or less	1204 (34.0)	351 (29.6)	645 (36.7)	208 (34.6)	0.002
Secondary/VT/Bachelor	1978 (55.8)	698 (58.9)	947 (53.9)	333 (55.3)	
University	363 (10.2)	136 (11.5)	166 (9.4)	61 (10.1)	
Ethnicity, N (%)					
Caucasian	3520 (99.2)	1180 (99.5)	1743 (99.1)	597 (99.0)	0.39
Other	28 (0.8)	6 (0.5)	16 (0.9)	6 (1.00)	
Civil status, N (%)					
Single	195 (5.5)	100 (8.4)	81 (4.6)	14 (2.4)	<0.001
Married	2849 (80.3)	929 (78.3)	1423 (80.9)	497 (82.4)	
Divorced	267 (7.5)	91 (7.7)	130 (7.4)	46 (7.6)	
Widow	238 (6.7)	67 (5.6)	125 (7.1)	46 (7.6)	
Location of the screening center, N (%)					
Corunna	529 (14.9)	171 (14.4)	265 (15.1)	93 (15.4)	<0.001
Barcelona	490 (13.8)	145 (12.2)	261 (14.8)	84 (13.9)	
Burgos	504 (14.2)	182 (15.3)	242 (13.7)	80 (13.3)	
Palma	535 (15.1)	226 (19.0)	239 (13.6)	70 (11.6)	
Pamplona	495 (13.9)	185 (15.6)	235 (13.3)	75 (12.4)	
Zaragoza	502 (14.1)	117 (9.9)	267 (15.2)	118 (19.6)	
Valencia	495 (14.0)	161 (13.6)	251 (14.3)	83 (13.8)	
Size of residence town, N (%)					
>200000 hab	879 (24.8)	342 (28.9)	395 (22.4)	142 (23.6)	<0.001
≤200000 hab	2667 (75.2)	841 (71.1)	1365 (77.6)	461 (76.4)	
Occupational status, N (%)					
Working	1507 (42.5)	528 (44.6)	729 (41.4)	250 (41.7)	0.02
Worked in the past	1535 (43.3)	516 (43.6)	749 (42.6)	270 (45.0)	
Never worked	502 (14.2)	140 (11.8)	282 (16.0)	80 (13.3)	
Eating out, N (%)					
No	1844 (52.1)	551 (46.5)	950 (54.3)	343 (57.0)	<0.001
Yes	1693 (47.9)	633 (53.5)	801 (45.7)	259 (43.0)	
Caring others at home, N (%)					
No	3284 (92.5)	1097 (92.4)	1634 (92.8)	553 (91.7)	0.65
Yes	266 (7.5)	90 (7.6)	126 (7.2)	50 (8.3)	
Family cancer, N (%)					
Yes	1624 (45.8)	531 (44.7)	831 (47.2)	262 (43.4)	0.45
No	1911 (53.8)	650 (54.8)	923 (52.4)	338 (56.1)	
NR/DK	15 (0.4)	6 (0.5)	6 (0.4)	3 (0.5)	
Deliveries					
None	318 (9.0)	153 (12.9)	140 (8.0)	25 (4.2)	<0.001
1–2	2245 (63.2)	766 (64.5)	1103 (62.7)	376 (62.3)	
>3	987 (27.8)	268 (22.6)	517 (29.3)	202 (33.5)	
Miscarriages/abortions, N (%)					
None	2791 (78.6)	913 (76.9)	1398 (79.4)	480 (79.6)	0.43
1	600 (16.9)	216 (18.2)	290 (16.5)	94 (15.6)	
≥2	159 (4.5)	58 (4.9)	72 (4.1)	29 (4.8)	
Caloric intake (Kcal/day), mean(SD)	2053.5 (479.8)	2149.4 (486.1)	2024.1 (479.0)	1951.2 (435.8)	<0.001
TOTAL	3550	1187 (33.4)	1760 (49.6)	603 (17.0)	

*DDM-Spain study: Determinants of Mammographic Density in Spain.

Abbreviations: SD = standard deviation; P = p value.

^**a**^Compliance withAdherence to European Code Against Cancer recommendations for cancer prevention.

^b^Pearson chi-square for categorical variables and Student’s t-test for continuous variables.

Table 2 shows the association of those sociodemographic, reproductive and lifestyle factors with the overall score of adherence (P < 0.10), adjusting only for age and screening center (simple model) and additionally including all of them in a final model (right columns). According to the geographical location of the screening center, women from Zaragoza showed a statistically significant higher adherence to ECAC recommendations, whereas women from Palma had the worst degree of adherence. Better adherence was observed in older women (OR_age≥60_:1.25; CI:1.07–1.45), in women who lived in rural and intermediate towns (OR:1.28; CI:1.09–1.50) and among those with more children (OR_≥3 children_:1.90; CI:1.39–2.58). Conversely, younger women, single, and those who consumed more calories showed lower adherence to cancer recommendations. We did not observe differences in global adherence by educational level in the multivariated model.

**Table 2:** Association between adherence to European Code Against cancer recommendations and potential influential factors.

Variables	Simple model				Final model^a^			
	N	OR^b^	CI 95%	P	N	OR^c^	CI 95%	P
Age								
45–49	1515	0.74	(0.61; 0.91)	0.01	1502	0.77	(0.62; 0.95)	0.01
50–59	1941	1.00	—	—	1930	1.00	—	—
60–68	94	1.39	(1.20; 1.60)	<0.001	93	1.25	(1.07; 1.45)	0.004
Educational level								
Primary or less	1204	1.00	—	—	1195	1.00	—	—
Secondary/ VT/Bachelor	1968	0.86	(0.74; 0.99)	0.04	1968	1.00	(0.86; 1.16)	0.99
University	363	0.85	(0.68; 1.08)	0.19	362	1.11	(0.87; 1.41)	0.42
Civil status								
Single	195	1.00	—	—	191	1.00	—	—
Married	2849	2.14	(1.62; 2.84)	<0.001	2830	1.46	(1.01; 2.11)	0.04
Divorced	267	2.19	(1.54; 3.13)	<0.001	266	1.55	(1.01; 2.37)	0.04
Widow	238	2.35	(1.63; 3.39)	<0.001	238	1.55	(1.00; 2.39)	0.05
Location of the screening center^d^								
Corunna	529	0.98	(0.84; 1.14)	0.80	525	0.90	(0.77; 1.06)	0.19
Barcelona	490	1.05	(0.90; 1.23)	0.56	488	1.06	(0.91; 1.25)	0.46
Burgos	504	1.00	(0.85; 1.17)	0.96	503	0.93	(0.78; 1.10)	0.40
Palma	535	0.66	(0.56; 0.77)	<0.001	533	0.73	(0.61; 0.86)	<0.001
Pamplona	495	0.93	(0.79; 1.09)	0.39	494	0.88	(0.75; 1.05)	0.15
Zaragoza	502	1.50	(1.29; 1.76)	<0.001	488	1.56	(1.33; 1.84)	<0.001
Valencia	495	1.06	(0.91; 1.24)	0.43	494	1.13	(0.96; 1.32)	0.14
Size of residence town								
>200000 hab	879	1.00	—	—	873	1.00	—	—
≤200000 hab	2667	1.36	(1.16; 1.58)	<0.001	2652	1.28	(1.09; 1.50)	0.002
Eating out								
No	1844	1.00	—	—	1837	1.00	—	—
Yes	1693	0.81	(0.71; 0.92)	0.001	1688	0.88	(0.77; 1.09)	0.07
Deliveries								
None	318	1.00	—	—	314	1.00	—	—
1–2	2245	1.87	(1.49; 2.34)	<0.001	2234	1.48	(1.10; 1.98)	0.01
≥3	987	2.40	(1.88; 3.07)	<0.001	977	1.90	(1.39; 2.58)	<0.001
Caloric intake (Kcal/day)								
<1812.6	1171	1.00	—	—	1163	1.00	—	—
1812.6–2212.6	1171	0.78	(0.67; 0.91)	0.002	1164	0.79	(0.67; 0.92)	0.003
>2212.6	1206	0.55	(0.46; 0.64)	<0.001	1198	0.54	(0.46; 0.64)	<0.001

Abbreviations: N = number of participants; OR = odds ratio; CI = confidence interval; P = p value.

^a^25 women were lost due to missing values.

^b^Adjusted for age and screening center.

^c^Adjusted for age, screening center, educational level, size of town, eating out, civil status, number of deliveries and caloric intake.

^d^Using the mean as the reference.

Tables 3–5 show the association of these characteristics with adherence to each specific ECAC recommendation. Table 3 includes recommendations related to tobacco, body weight and physical activity; Table 4 presents the three recommendations related with a healthy diet and Table 5 shows the last three: alcohol, breastfeed and HRT use. Due to the small number of women who did not adhere to the “limit high-calorie foods and sugary drinks” recommendation, the first two categories (non adherent and partially adherent women) of this variable were combined in a single group, which was used as a reference. While older women showed higher adherence to recommendations related to smoking and breastfeeding, these same women presented worse adherence to recommendations related to body weight and physical activity. Women with higher educational level seemed to have greater adherence to recommendations related to maintaining a healthy weight, but they adhered worse to the recommendations related to alcohol and HRT use. Women living in rural or intermediate towns had higher adherence to recommendations related to tobacco, physical activity and consumption of high calorie foods and sugary drinks. Women who reported eating out several times per month had higher adherence to the recommendation of being physically active, but failed to limit their alcohol intake and the consumption of red/processed meat and salty food. Parous women drank less alcohol, but were less prone to maintain a healthy body weight and to limit the consumption of high calorie foods, sugary drinks, red/processed meat and salty food. Furthermore, compared to women with 1–2 children, those with 3 or more children had greater adherence to the recommendation of breastfeeding, as expected. On the other hand, while divorced women reported better adherence to maintaining a healthy weight, widows ate larger amounts of grains, pulses, vegetables and fruits and married and divorced women ate less quantity of high-calorie foods and sugary drinks. Finally, women who consumed more calories had a healthier weight and did more physical activity, but also consumed more grains, pulses, vegetables, fruits, high-calorie foods, sugary drinks, red and processed meat, salty food and alcohol.

**Table 3:** Association between adherence to smoking, weight and physical activity recommendations and potential influential factors.

Variables	N	Don’t smoke			Be a healthy body weight			Be physically active		
		OR^a,b^	CI 95%	P	OR^a,b^	CI 95%	P	OR^a,b^	CI 95%	P
Age										
45–49	1502	0.41	(0.32; 0.52)	<0.001	1.71	(1.35; 2.17)	<0.001	0.88	(0.66; 1.17)	0.37
50–59	1930	1.00	—	—	1.00	—	—	1.00	—	—
60–68	93	2.57	(2.01; 3.28)	<0.001	0.68	(0.56; 0.83)	<0.001	0.75	(0.62; 0.90)	0.002
Educational level										
Primary or less	1195	1.00	—	—	1.00	—	—	1.00	—	—
Secondary/ VT/Bachelor	1968	0.81	(0.65; 1.00)	0.05	1.55	(1.28; 1.89)	<0.001	1.24	(1.03; 1.49)	0.02
University	362	0.81	(0.59; 1.10)	0.17	2.60	(1.97; 3.44)	<0.001	1.06	(0.78; 1.43)	0.71
Civil status										
Single	191	1.00	—	—	1.00	—	—	1.00	—	—
Married	2830	1.53	(0.98; 2.39)	0.06	1.08	(0.71; 1.64)	0.72	1.33	(0.86; 2.05)	0.20
Divorced	266	0.77	(0.47; 1.28)	0.32	1.79	(1.11; 2.89)	0.02	0.94	(0.57; 1.55)	0.82
Widow	238	1.00	(0.58; 1.73)	0.99	0.81	(0.48; 1.37)	0.44	1.22	(0.73; 2.05)	0.45
Location of the screening center^c^										
Corunna	525	1.03	(0.82; 1.28)	0.82	1.15	(0.95; 1.40)	0.15	1.55	(1.24; 1.93)	<0.001
Barcelona	488	1.17	(0.92; 1.48)	0.20	0.68	(0.55; 0.84)	<0.001	0.55	(0.46; 0.67)	<0.001
Burgos	503	0.90	(0.72; 1.13)	0.36	1.35	(1.11; 1.64)	0.003	2.60	(1.95; 3.45)	<0.001
Palma	533	0.84	(0.67; 1.04)	0.11	1.05	(0.85; 1.28)	0.67	0.73	(0.60, 0.88)	0.001
Pamplona	494	0.88	(0.70; 1.09)	0.23	1.21	(0.99; 1.47)	0.07	0.62	(0.50; 0.75)	<0.001
Zaragoza	488	0.94	(0.75; 1.17)	0.56	1.38	(1.14; 1.68)	0.001	0.83	(0.68; 1.01)	0.07
Valencia	494	1.35	(1.08; 1.69)	0.01	0.54	(0.44; 0.67)	<0.001	1.21	(0.98; 1.50)	0.08
Size of residence town										
>200000 hab	873	1.00	—	—	1.00	—	—	1.00	—	—
≤200000 hab	2652	1.47	(1.20; 1.81)	<0.001	0.97	(0.80; 1.17)	0.74	1.26	(1.04; 1.52)	0.02
Eating out										
No	1837	1.00	—	—	1.00	—	—	1.00	—	—
Yes	1688	0.87	(0.73; 1.04)	0.14	1.13	(0.96; 1.32)	0.15	1.35	(1.14; 1.59)	0.001
Deliveries										
None	314	1.00	—	—	1.00	—	—	1.00	—	—
1–2	2234	1.09	(0.76; 1.56)	0.65	0.65	(0.47; 0.91)	0.01	1.25	(0.88; 1.77)	0.21
≥3	977	1.17	(0.79; 1.74)	0.44	0.44	(0.31; 0.64)	<0.001	1.12	(0.77; 1.63)	0.55
Caloric intake (Kcal/day)										
<1812.6	1163	1.00	—	—	1.00	—	—	1.00	—	—
1812.6–2212.6	1164	1.03	(0.83; 1.27)	0.80	1.24	(1.02; 1.51)	0.03	1.43	(1.17; 1.75)	<0.001
>2212.6	1198	1.01	(0.81; 1.25)	0.95	1.42	(1.16; 1.73)	0.001	1.43	(1.17; 1.76)	0.001

Abbreviations: N = number of participants; OR = odds ratio; CI = confidence interval; P = p value.

^a^Adjusted for age, screening center, educational level, size of town, eating out, civil status, number of deliveries, caloric intake and the overall adherence to the other recommendations.

^b^25 women were lost due to missing values.

^c^Using the mean as the reference.

**Table 4:** Association between adherence to diet recommendations and potential influential factors.

Variables	N	Eat plenty of grains, pulses, vegetables and fruits			Limit high-calorie foods and sugary drinks			Limit red/processed meat and salty food		
		OR^a,b^	CI 95%	P	OR^a,b^	CI 95%	P	OR^a,b^	CI 95%	P
Age										
45–49	1502	1.56	(0.63; 3.84)	0.34	0.95	(0.34; 2.68)	0.92	0.75	(0.59; 0.95)	0.02
50–59	1930	1.00	—	—	1.00	—	—	1.00	—	—
60–68	93	1.24	(0.71; 2.15)	0.45	1.00	(0.34; 2.92)	0.99	1.10	(0.93; 1.30)	0.25
Educational level										
Primary or less	1195	1.00	—	—	1.00	—	—	1.00	—	—
Secondary/ VT/Bachelor	1968	0.91	(0.52; 1.59)	0.74	0.83	(0.29; 2.39)	0.73	0.80	(0.68; 0.95)	0.01
University	362	2.37	(0.83; 6.78)	0.11	0.63	(0.14; 2.85)	0.55	0.83	(0.64; 1.09)	0.18
Civil status										
Single	191	1.00	—	—	1.00	—	—	1.00	—	—
Married	2830	2.56	(0.91; 7.23)	0.08	8.73	(1.72; 44.4)	0.01	1.12	(0.74; 1.68)	0.60
Divorced	266	1.69	(0.51; 5.57)	0.39	8.20	(0:98; 68.57)	0.05	1.37	(0.86; 2.20)	0.19
Widow	238	6.86	(1.249; 37.79)	0.03	4.73	(0.58; 39.45)	0.15	1.31	(0.81; 2.14)	0.28
Location of the screening center^c^										
Corunna	525	0.39	(0.25; 0.62)	<0.001	2.27	(0.39; 13.41)	0.36	0.85	(0.71; 1.02)	0.08
Barcelona	488	0.63	(0.36; 1.12)	0.12	1.10	(0.36; 3.40)	0.89	0.86	(0.72; 1.03)	0.11
Burgos	503	1.17	(0.56; 2.46)	0.68	0.27	(0.10; 0.71)	0.01	1.03	(0.85; 1.25)	0.77
Palma	533	3.39	(1.20; 9.61)	0.02	1.13	(0.39; 3.29)	0.83	1.11	(0.92; 1.34)	0.27
Pamplona	494	0.84	(0.43; 1.64)	0.62	1.01	(0.31; 3.31)	0.99	0.88	(0.73; 1.06)	0.18
Zaragoza	488	1.37	(0.64; 2.93)	0.42	2.64	(0.45; 15.49)	0.28	1.12	(0.94; 1.35)	0.21
Valencia	494	0.88	(0.45; 1.73)	0.72	0.49	(0.20; 1.20)	0.12	1.21	(1.02; 1.45)	0.04
Size of residence town										
>200000 hab	873	1.00	—	—	1.00	—	—	1.00	—	—
≤200000 hab	2652	1.22	(0.71; 2.11)	0.47	2.59	(0.97; 6.91)	0.06	1.00	(0.84; 1.19)	0.99
Eating out										
No	1837	1.00	—	—	1.00	—	—	1.00	—	—
Yes	1688	1.00	(0.62; 1.63)	0.99	1.50	(0.65; 3.46)	0.35	0.87	(0.75; 1.01)	0.07
Deliveries										
None	314	1.00	—	—	1.00	—	—	1.00	—	—
1–2	2234	0.88	(0.33; 2.36)	0.80	0.75	(0.55; 1.02)	0.07	0.70	(0.51; 0.98)	0.04
≥3	977	0.90	(0.30; 2.69)	0.86	0.71	(0.51; 0.99)	0.04	0.69	(0.49; 0.99)	0.04
Caloric intake (Kcal/day)										
<1812.6	1163	1.00	—	—	1.00	—		1.00	—	—
1812.6–2212.6	1164	3.34	(1.83; 6.12)	<0.001	0.46	(0.09; 2.41)	0.36	0.46	(0.39; 0.55)	<0.001
>2212.6	1198	5.00	(2.48; 10.09)	<0.001	0.14	(0.03; 0.62)	0.01	0.17	(0.14; 0.21)	<0.001

Abbreviations: N = number of participants; OR = odds ratio; CI = confidence interval; P = p value.

^a^Adjusted for age, screening center, educational level, size of town, eating out, civil status, number of deliveries, caloric intake and the overall adherence to the other recommendations.

^b^25 women were lost due to missing values.

^c^Using the mean as the reference.

**Table 5:** Association between adherence to alcohol, breastfeeding and hormone replacement therapy use recommendations and potential influential factors.

Variables	N	Limit your alcohol intake			Breastfeed your baby			Limit HRT use		
		OR^a,b^	CI 95%	P	OR^a,b^	CI 95%	P	OR^a,b^	CI 95%	P
Age										
45–49	1502	1.99	(1.25; 3.17)	0.004	0.98	(0.77; 1.24)	0.85	1.67	(0.99; 2.83)	0.06
50–59	1930	1.00	—	—	1.00	—	—	1.00	—	—
60–68	93	0.84	(0.57; 1.25)	0.40	1.31	(1.10; 1.56)	0.002	0.75	(0.52; 1.08)	0.13
Educational level										
Primary or less	1195	1.00	—	—	1.00	—	—	1.00	—	—
Secondary/ VT/Bachelor	1968	0.75	(0.48; 1.15)	0.18	0.97	(0.82; 1.15)	0.71	0.62	(0.41; 0.93)	0.02
University	362	0.57	(0.32; 1.04)	0.07	1.01	(0.76; 1.35)	0.92	0.63	(0.34; 1.16)	0.14
Civil status										
Single	191	1.00	—	—	1.00	—	—	1.00	—	—
Married	2830	0.49	(0.20; 1.19)	0.11	2.21	(0.96; 5.06)	0.06	1.48	(0.44; 4.00)	0.45
Divorced	266	0.62	(0.21; 1.84)	0.39	1.94	(0.81; 4.60)	0.14	1.41	(0.46; 4.38)	0.55
Widow	238	0.43	(0.15; 1.26)	0.12	2.30	(0.96; 5.50)	0.06	0.98	(0.32; 2.97)	0.97
Location of the screening center^c^										
Corunna	525	2.05	(1.09; 3.88)	0.03	1.40	(0.86; 1.25)	0.69	1.35	(0.88; 2.07)	0.17
Barcelona	488	2.48	(1.32; 4.65)	0.01	1.61	(1.34; 1.94)	<0.001	1.47	(0.93; 2.33)	0.10
Burgos	503	0.22	(0.16; 0.32)	<0.001	0.89	(0.73; 1.08)	0.24	0.61	(0.42; 0.88)	0.01
Palma	533	0.58	(0.39; 0.85)	0.01	0.58	(0.48; 0.70)	<0.001	0.79	(0.53; 1.18)	0.25
Pamplona	494	0.54	(0.36; 0.81)	0.003	0.97	(0.80; 1.17)	0.73	0.68	(0.46; 0.99)	0.04
Zaragoza	488	1.72	(0.96; 3.07)	0.07	1.39	(1.15; 1.68)	0.001	1.49	(0.93; 2.40)	0.10
Valencia	494	1.66	(0.98; 2.82)	0.06	0.87	(0.72; 1.04)	0.12	1.04	(0.68; 1.58)	0.87
Size of residence town										
>200000 hab	873	1.00	—	—	1.00	—	—	1.00	—	—
≤200000 hab	2652	0.95	(0.60; 1.51)	0.83	1.09	(0.91; 1.30)	0.36	0.96	(0.63; 1.47)	0.86
Eating out										
No	1837	1.00	—	—	1.00	—	—	1.00	—	—
Yes	1688	0.65	(0.48; 0.93)	0.02	0.91	(0.78; 1.06)	0.21	0.96	(0.69; 1.33)	0.80
Deliveries										
None	314	1.00	—	—	—	—	—	1.00	—	—
1–2	2234	1.76	(0.94; 3.29)	0.08	1.00	—	—	0.48	(0.20; 1.18)	0.11
≥3	977	2.17	(1.09; 4.32)	0.03	2.72	(2.30; 3.21)	<0.001	0.58	(0.23; 1.49)	0.26
Caloric intake (Kcal/day)										
<1812.6	1163	1.00	—	—	1.00	—		1.00	—	—
1812.6–2212.6	1164	0.49	(0.30; 0.80)	0.01	0.98	(0.82; 1.18)	0.83	1.05	(0.70; 1.56)	0.83
>2212.6	1198	0.28	(0.18; 0.46)	<0.001	1.12	(0.93; 1.35)	0.23	1.02	(0.68; 1.53)	0.93

Abbreviations: N = number of participants; OR = odds ratio; CI = confidence interval; P = p value.

^a^Adjusted for age, screening center, educational level, size of town, eating out, civil status and number of deliveries and the overall adherence to the other recommendations.

^b^25 women were lost due to missing values.

^c^Using the mean as the reference.

There were significant differences in the degree of adherence to these recommendations by geographical area. The greatest heterogeneity was observed for consumption of grains, pulses, vegetables and fruits (high adherence in Palma and low in Corunna), the limitation in alcohol consumption (high adherence in Corunna and Barcelona and low in Burgos) and the limitation in the consumption of high-calorie foods and sugary drinks (high adherence in Zaragoza and Corunna and low in Burgos). Maintaining a healthy body weight was mainly observed in Zaragoza and Burgos. This last city was also the one with greater adherence to the recommendation of being physically active. Valencia was the city that consumed the least amount of red/processed meat and salty food. Palma had very poor adherence to the breastfeeding recommendation. Burgos and Pamplona were the most HRT users. Finally, differences in smoking were less marked.

## Discussion

Although in recent years many studies have explored the association of the adherence to cancer prevention guidelines with cancer risk or mortality^25–27^, very few have explored the sociodemographic and lifestyle factors influencing such adherence^28^. The present study tries to know how Spanish women behave with respect to cancer risk factors, taking as a reference the recommendations of the ECAC. The study also tries to identify which sociodemographic and lifestyle factors are conditioning the acquisition of healthier lifestyles, in order to intervene in them with public health actions. Our results show that global adherence to these recommendations by Spanish women attending breast cancer screening is moderate, higher for HRT use, alcohol intake and consumption of vegetables, fruits and grains, and lower for consumption of processed meat, red meat and foods high in salt, physical activity and body weight. In general, overall adherence was higher among older women, parous women and in those living in rural areas. Our results also highlight differences in adherence to specific recommendations according to women’s characteristics, which should inform prevention campaigns. Thus, older women should increase physical activity and take better care of their body weight, while younger women should stop smoking as well as decrease the intake of red and processed meat and salty products. Women who eat out frequently should give attention to their consumption of alcohol and red/processed meat and salty food. Women with children should also reduce the intake of this type of food, as well as high calorie food and sugary drinks, and take better care of their body weight. Finally, women with higher caloric intake should reduce the consumption of the mentioned products, as well as their alcohol intake. The adherence to recommendations was very heterogeneous in the different geographical locations, being Zaragoza and Palma the cities with healthier and unhealthier lifestyles respectively.

Our study has some limitations that must be taken into account. Firstly, we could not evaluate other recommendations included in the ECAC for cancer prevention due to lack of information on passive smoking, sun and sunbeds exposure, occupational exposure to carcinogens and use of protective equipment, natural radiation and status of vaccination against hepatitis B (HPV vaccination has been introduced recently in our country). Secondly, with the exception of body mass index (BMI), the information on women characteristics was self-reported, so behaviors considered socially desirable or healthy may have been overestimated by our participants, consciously or unconsciously. Regarding the representativeness of the study population, it has been argued that women attending screening programs may have different sociodemographic characteristics and tend to be more aware of the importance of prevention than the general population^38^. However, coverage of breast cancer screening in Spain reached almost 100% of the target population in all Autonomous Communities in 2006, with high participation rates^39^, which supports the external validity of our results. In addition, according to data provided by the National Health Survey for Spanish women in the same age range^40^, our women were very similar to the national sample in terms of prevalence of alcohol consumption, prevalence of non-smokers, prevalence of intense physical activity and use of hormone replacement therapy.

The relative independence of each ECAC recommendation justify the discussion of our results separately.

### Don’t smoke

Tobacco smoking is the single largest preventable cause of cancer in the European Union. Although Spain is one of the European countries with higher proportion of smokers^41^, a reduction has been observed following the implementation of the last smoke-free legislation. In 2011, the prevalence of current smokers (daily and casual) in adult women was 22.8%^40^, very similar to the data reported here.

Adherence to tobacco recommendation was higher in older women, in less educated women and in those living in middle-sized towns and rural areas. Smoking prevalence in Spanish females varied greatly by birth cohort and education. The tobacco epidemic started earlier among highly educated women, and those born between 1960 and 1980 were exposed to large amounts of targeted advertisement at a vulnerable age^42^. These generations are represented in our study by those women younger than 50. Currently, although prevalence has declined at all levels, it is still higher in women with higher education^40^. Regarding population size, Idris *et al*.^43^ also reported higher prevalence of smoking in women living in urban areas of Spain, being these inequalities more pronounced among those with low educational level.

### Be a healthy body weight

Body fatness is an important problem in Spain, where the prevalence of overweight and obesity have increased progressively, reaching a rate of 24.1% of obese women and 54.6% of overweight women in 2014^44^. Given these figures, it is not strange to find that this recommendation shows the lowest level of adherence (71% of no adherence). Older women and those with more deliveries presented higher BMI in our study. The association of obesity with age is well known^45^, and the increased BMI associated with increasing parity (independently of socioeconomic and other lifestyle factors) has also been previously described^46^. On the other hand, women with higher educational level tended to present healthier BMI. Rodriguez Caro *et al*.^47^, in a recent study, described that education was the most important source of social differences in obesity among women in Spain, being this educational gradient greater at the end of the BMI distribution.

### Be physically active

There is strong evidence that physical activity protects against colon, breast and endometrial cancer, and probably also against lung cancer^10,12,15^. In Spain, the prevalence of physical inactivity is increasing, reaching a rate of 44% in 2013^48^.

In our study, the proportion of women who classified themselves as mostly inactive was 24%, which is lower than that reported by the National Health Survey in relation to adherence to the World Health Organization recommendations for physical activity (35.8%)^49^. The adherence of our women differed by age and size of town. The greater frequency of physical activity in younger people has been described in Europe^48^, and Whelan *et al*.^28^ also described greater adherence to the physical activity recommendation among this collective. The higher prevalence of physical activity among women living in rural areas could be related to regular walking, since Spain is the second country of the European Union where regular walking is more prevalent^48^, and the rural areas are a favourable environment for this type of activity.

*Have a healthy diet*: *Recommendations on* (1) *increasing intake of whole grains*, *pulses*, *vegetables and fruits*, (2) *limiting*/*avoiding high*-*calorie foods and sugary drinks*, (3) *avoiding*/*limiting processed meat*, *meat and foods high in salt*

We have evaluated these recommendations separately because the degree of adherence is very heterogeneous in our population. Although most women meet the recommendation of high consumption of fruits and vegetables, adherence to the recommendation to limit consumption of red and processed meat, fast food, and salty foods has been very low, mainly among younger women. Regarding fast food, high-calorie foods and sugary drinks, the percentage of women with lack of adherence was very low, except in the case of fast food. In fact, the consumption and the variety of prepared dishes has risen significantly in recent years in Spanish households^50^ and, as in our study, the demand for these products increases with the size of the population^51^. The consumption of meat products in 2009 accounted for 20.8% of total food expenditure^52^, and 21.8% in 2015^53^. The demand for these products outside the home experienced a significant increase from 1995 to 2009, retreating subsequently due to the crisis^52^. As in our study, middle-upper-class households showed the highest consumption. However, contrary to what was observed in 2009 in Spanish households^52^, the parous women of our study showed a higher demand for these products.

### Alcohol

It has been estimated that about 4.2% of all cancer cases in women are attributable to alcohol consumption in the European Region^54^. In Spain, this figure is around 4%^55^. In our study, more than 80% of women totally accomplished this recommendation, which is in consonance with the considerable decrease in alcohol intake observed in the last decade^56,57^. Adherence to this recommendation was higher among younger women and lower among those with a university degree and among those who reported eat out frequently and consumed more calories. The latest alcohol survey in Spain^58^ also shows that, in general, alcohol consumption has shifted towards older women, with greater prevalence among women aged 55–64. However, the highest prevalence is observed in adolescents and young adults, women between 15 and 24 years old^58^. On the other hand, alcohol consumption in Spanish women is considerably higher at the highest educational levels: 71.7% of university women had drunk alcohol in the last year, compared to 27.9% of women without studies^58^.

### Breastfeeding

In Spain, the percentage of children under 5 who have been totally or partially breastfed has increased since 1995 thanks to the Initiative for the Humanization of Birth and Breastfeeding Assistance (IHAN) (launched by WHO and UNICEF to encourage hospitals, health services, and maternity wards to adopt practices that promote and support exclusive breastfeeding from birth)^59^. According to the latest National Health Survey, 66.5% of infants were exclusively or partially breastfed up to 3 months of age, and 47.0% up to 6 months in 2011^59^. In our study, older women presented higher adherence to this recommendation, which might be due to a higher proportion of housewives at time of delivery, compared to younger cohorts, which facilitated the extension of the lactation period. Obviously, multiparous women comply better with this recommendation, which includes life-long accumulated breastfeeding. Moreover, a previous successful experience with breastfeeding can also make women more prone to breastfeed the next child.

### Hormonal replacement therapy

Spain is one of the European countries with the lowest HRT use^60^, which has declined progressively from 2001 to 2007, but remains stable since then^61^. It has been estimated that the prevalence of current HRT use in 2014 was 0.21% in women older than 40 years, with the 40–45 age group being the most prevalent^61^. This recommendation shows the highest degree of adherence in our study (8.6% ever used HRT). Our rate is similar to the prevalence described in another population-based Spanish multicenter study (9.8%) in which HRT use was associated with higher social class^62^. This study, also reported that women born in the 1940s (older women of our study) were the most exposed to HRT^62^.

In conclusion, our results show that the degree of adherence to the lifestyle ECAC recommendations for cancer prevention among Spanish women attending breast cancer screening was, in general, moderate. Two out of three women adhered totally or partially. Some recommendations should be encouraged, particularly those related to obesity, meat and salty food consumption and breastfeeding. In Spain, where most people never heard about ECAC recommendations, outreach campaigns targeting both the general public and health professionals are needed, as well as government policies and actions that facilitate healthier choices and create environments that promote behavioral change towards healthier lifestyles. The high participation rates in the Spanish breast cancer screening programmes reflect the interest of these women in cancer prevention. This may be a critical moment to provide information on lifestyles useful to decrease their risk of cancer. It would also be very interesting that the National Strategy in Cancer periodically evaluate the degree of adherence to cancer prevention recommendations defined in the ECAC in different population areas, in order to measure the effectiveness of this tool promoted by IARC.

## Supplementary information

Operationalization of the European Code Against Cancerrecommendations for cancer prevention in a score

## Data Availability

The datasets generated are not publicly available due to restrictions imposed by the Carlos III Ethic Committee, but are available from the principal investigator on reasonable request.
