# Supplementary material for: Prevalence of healthy lifestyles against cancer in Spanish women
Source: Sci Rep. 2019 Jul 23;9:10638. doi: 10.1038/s41598-019-47180-x (PMC6650391; doi:10.1038/s41598-019-47180-x)
Supplement: Supplementary file 1 — Operationalization of the European Code Against Cancerrecommendations for cancer prevention in a score [file 41598_2019_47180_MOESM1_ESM.docx]

**Prevalence of healthy lifestyles against cancer in Spanish women**

María José Toribio, Virginia Lope, Adela Castelló, Dolores Salas, Carmen Vidal, Nieves Ascunce, Carmen Santamariña, Pilar Moreo, Carmen Pedraz-Pingarrón, Carmen Sánchez-Contador, Nuria Aragonés, Beatriz Pérez-Gómez, Marina Pollán

**Table S1**. Operationalization of the European Code Against Cancer recommendations for cancer prevention in a score

| **Recommendation** | **Source** | **Operationalization** | **Scoring** |
| --- | --- | --- | --- |
| **Do not smoke. Do not use any form of tobacco.** | Smoking along the life (self-reference) | Smoker | 0 |
|  |  | Former smoker | 0.5 |
|  |  | Never smoker | 1 |
| **Take action to be a healthy body weight.** | BMI calculated from anthropometric measures taken | BMI≥25 kg/m^2^ | 0 |
|  | by interviewers | BMI<25 kg/m^2^ | 1 |
| **Be physically active in everyday life. Limit the time you spend sitting.** | Sports and activity at workplace/at home (self-reference) | Sitting at work/scarce at-home activity **^a^** | 0 |
|  |  | Mild sport activity/significant at-home activity **^a^** | 0.5 |
|  |  | High performance sports/physical work **^a^** | 1 |
| **Have a healthy diet: Eat plenty of whole grains, pulses, vegetables and fruits** | Average of (a) and (b) below | Low adherence | <0.33 |
|  |  | Moderate | 0.33-0.67 |
|  |  | High adherence | >0.67 |
| (a) Vegetables and fruits ^d^ | Last year intake (self-reference) | <200 g/d **^a^** | 0 |
|  |  | 200-<400 g/d **^a^** | 0.5 |
|  |  | ≥400 g/d **^a^** | 1 |
| (b) Wholegrain and pulses ^e^ | Last year intake (self-reference) | < 20 g/d **^b^** | 0 |
|  |  | 20-<60 g/d **^b^** | 0.5 |
|  |  | ≥60 g/d **^b^** | 1 |
| **Have a healthy diet: Limit high-calorie foods and avoid sugary drinks** | Average of (c), (d) and (e) below | Low adherence | <0.33 |
|  |  | Moderate | 0.33-0.67 |
|  |  | High adherence | >0.67 |
| (c) Fast food ^f^ | Last year intake (self-reference) | ≥15 g/d **^b^** | 0 |
|  |  | 7-<15 g/d **^b^** | 0.5 |
|  |  | < 7 g/d **^b^** | 1 |
| (d) Foods high in sugar and fat ^g^ | Last year intake (self-reference) | ≥175 Kcal/100 g **^b^** | 0 |
|  |  | 125-<175 Kcal/100g **^b^** | 0.5 |
|  |  | <125 Kcal/100 g **^b^** | 1 |
| (e) Sugary drinks ^h^ | Last year intake (self-reference) | > 250 g/d **^b^** | 0 |
|  |  | Up to 250 g/d **^b^** | 0.5 |
|  |  | No intake **^b^** | 1 |
| **Have a healthy diet: Avoid processed meat, limit read meat and foods high in salt** | Average of (f) and (g) below | Low adherence | <0.33 |
|  |  | Moderate | 0.33-0.67 |
|  |  | High adherence | >0.67 |
| (f) Red and processed meat ^i^ | Last year intake (self-reference) | >500 g/wk **^b^** | 0 |
|  |  | 300-500 g/wk **^b^** | 0.5 |
|  |  | <300 g/wk **^b^** | 1 |
| (g) Food high in salt ^j^ | Last year intake (self-reference) | > 30 g/d **^b^** | 0 |
|  |  | 21-30 g/d **^b^** | 0.5 |
|  |  | <21 g/d **^b^** | 1 |
| **If you drink alcohol of any type, limit your intake. Not drinking alcohol is better for cancer prevention ^k^** | Last year intake (self-reference) | >20 g/d **^a^** | 0 |
|  |  | 10-20 g/d **^a^** | 0.5 |
|  |  | <10 g/d | 1 |
| **Breaksfeeding reduces the mother's cancer risk. If you can, breastfeed your baby (only for women who have had children)** | Average of (h) and (i) below | Low adherence | <0.33 |
|  |  | Moderate | 0.33-0.67 |
|  |  | High adherence | >0.67 |
| (h) Having breasteed a child at least 6 months |  | No ^c^ | 0 |
|  |  | Yes ^c^ | 1 |
| (i) Cumulative breastfeeding periods along life |  | <1 months ^c^ | 0 |
|  |  | 1-6 months ^c^ | 0.5 |
|  |  | >6 months ^c^ | 1 |
| **Hormone replacement therapy (HRT) increases the risk of certain cancers; limit use of HRT (only for postmenopausal women)** |  | >5 years | 0 |
|  |  | 5 years | 0.5 |
|  |  | No | 1 |

^a^ Scoring obtained from World Cancer Research Fund and American Institute for Cancer Research Lifestyle Recommendations: EpiGEICAM Case-Control Study. PLoS One. 2015; 10(5):e0126096.

^b^ Scoring obtained from World Cancer Research Fund : Food, nutrition, physical activity and the prevention of cancer: a global perspective: A project of World Cancer Research Fund International. American Institute for Cancer Research; 2007.

^c^ Scoring obtained from European Code Against Cancer: 12 ways to reduce your Cancer Risk. International Agency for Research on Cancer; 2014.

^d^ Orange, mandarin, banana, apple, pear, peach, nectarine, apricot, watermelon, melon, grapes, plums or prunes (dried or fresh), kiwi, spinach, chard, lettuce, endive, escarole, tomato, eggplant, zucchini, cucumber, pepper, artichoke, carrot, pumpkin, cooked cabbage, cauliflower or broccoli, onion, green beans, asparagus, corn and garlic.

^e^ Whole-grain bread and partial whole-grain bread, breakfast cereals and legumes.

^f^ Chips, crisps, pizza, fish fingers, mayonnaise, ketchup.

^g^ Energy intake for all foods considered.

^h^ Sugary drinks: juices and other sugared beverages.

^i^ Red and processed meat: Pork, beef, lamb, liver (beef, pork or chicken), entrails, hamburger, cold meat, sausages, bacon, pâte, foie-gras.

^j^ Jarmon and other cold meat and smoked and salt preserved fish.

^k^ Wine, beer and spirit.
